# Supplementary material for: Multi-Omics Profiling of Human Endothelial Cells from the Coronary Artery and Internal Thoracic Artery Reveals Molecular but Not Functional Heterogeneity
Source: Int J Mol Sci. 2023 Oct 9;24(19):15032. doi: 10.3390/ijms241915032 (PMC10573276; doi:10.3390/ijms241915032)
Supplement: Supplementary file 1 [file ijms-24-15032-s001.zip › Legend to Supplementary Figure S1.pdf]

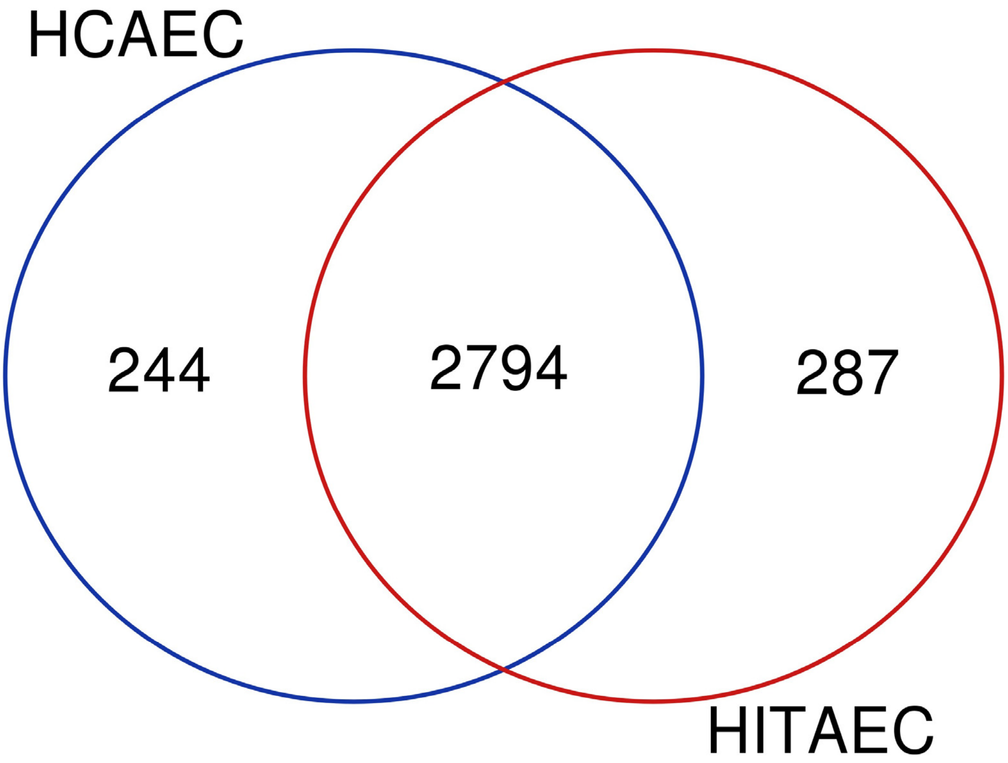

Supplementary Figure S1. Venn diagram showing the number of differentially expressed proteins (DEPs) in the proteome of human coronary artery endothelial cells (HCAEC, 244 DEPs) and human internal thoracic artery endothelial cells (HITAEC, 286 DEPs). DEPs were defined as those with logarithmical fold change ≥ 1 and FDR-corrected p value ≤ 0.05.
