# Supplementary material for: Multi-Omics Profiling of Human Endothelial Cells from the Coronary Artery and Internal Thoracic Artery Reveals Molecular but Not Functional Heterogeneity
Source: Int J Mol Sci. 2023 Oct 9;24(19):15032. doi: 10.3390/ijms241915032 (PMC10573276; doi:10.3390/ijms241915032)
Supplement: Supplementary file 1 [file ijms-24-15032-s001.zip › Legend to Supplementary Figure S3.pdf]

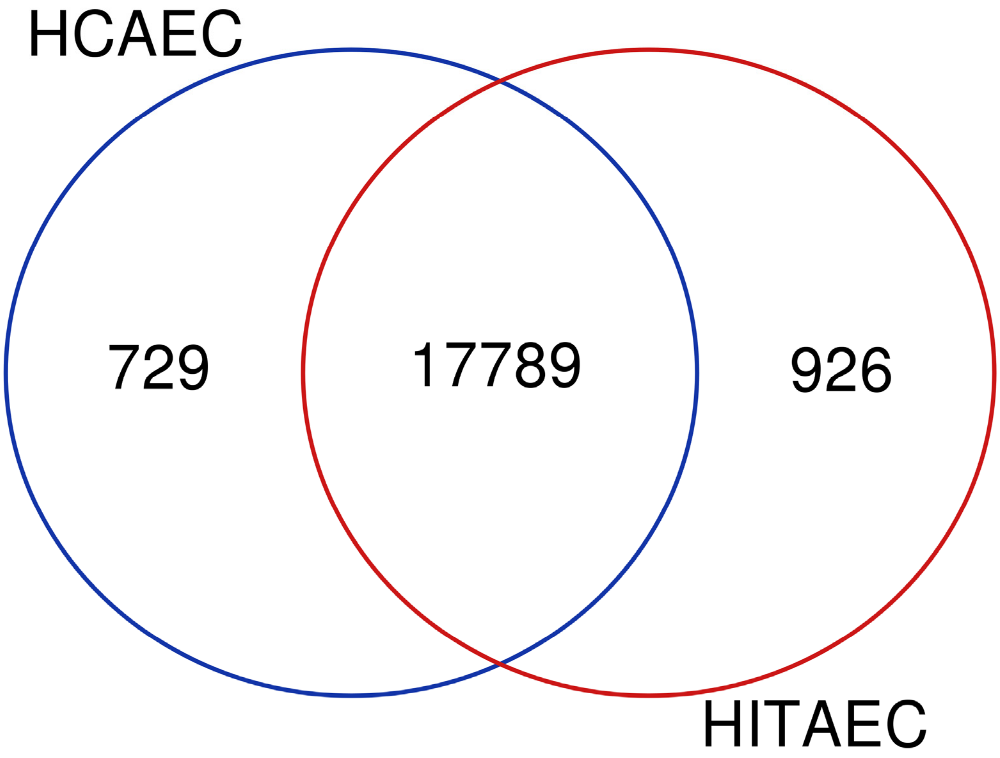

Supplementary Figure S3. Venn diagram showing the number of differentially expressed genes in the transcriptome of human coronary artery endothelial cells (HCAEC, 729 DEGs) and human internal thoracic artery endothelial cells (HITAEC, 926 DEGs) at static cell culture conditions. DEGs were defined as those with logarithmical fold change ≥ 1 and FDR-corrected p value ≤ 0.05.
