# Supplementary material for: Multi-Omics Profiling of Human Endothelial Cells from the Coronary Artery and Internal Thoracic Artery Reveals Molecular but Not Functional Heterogeneity
Source: Int J Mol Sci. 2023 Oct 9;24(19):15032. doi: 10.3390/ijms241915032 (PMC10573276; doi:10.3390/ijms241915032)
Supplement: Supplementary file 1 [file ijms-24-15032-s001.zip › Legend to Supplementary Figure S4.pdf]

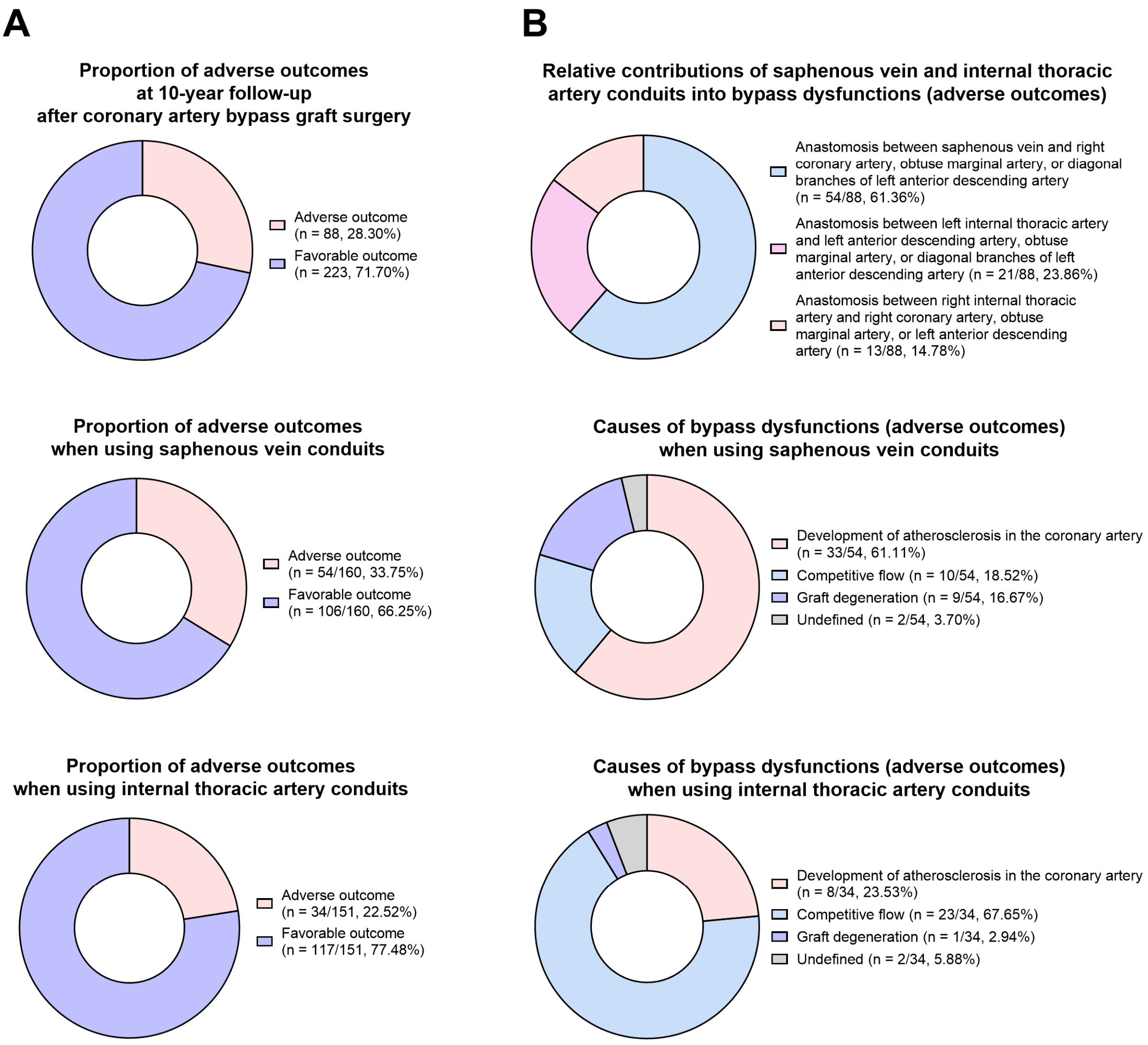

Supplementary Figure S4. Retrospective analysis of repeated coronary angiography performed in 102 patients (n = 311 bypasses) at 10 years of follow-up after coronary artery bypass graft surgery. **(a)** Average proportion of adverse outcomes (top) and proportions of adverse outcomes when using SV conduits (middle) and ITA conduits (bottom); **(b)** Analysis of bypass dysfunctions (i.e., adverse outcomes): relative contributions of SV and ITA conduits into the total number of bypass dysfunctions (top) and causes of bypass dysfunctions when using SV conduits (middle) and ITA conduits (bottom).
