# Supplementary material for: Multi-Omics Profiling of Human Endothelial Cells from the Coronary Artery and Internal Thoracic Artery Reveals Molecular but Not Functional Heterogeneity
Source: Int J Mol Sci. 2023 Oct 9;24(19):15032. doi: 10.3390/ijms241915032 (PMC10573276; doi:10.3390/ijms241915032)
Supplement: Supplementary file 1 [file ijms-24-15032-s001.zip › Supplementary Tables (S1-S40).pdf]

SUPPLEMENTARY TABLES

PROTEOMIC PROFILING

Supplementary Table S1. Molecular terms (selected from Gene Ontology (GO), UniProtKB Keywords, Reactome, and Kyoto Encyclopedia of Genes and Genomes (KEGG) databases) common for human coronary artery endothelial cells (HCAEC) and human internal thoracic artery endothelial cells (HITAEC) according to the proteomic profiling and pathway enrichment analysis. Gene Ontology database has been screened by cellular components (CC), molecular function (MF), and biological process (BP) domains.

Molecular term	Total proteins	Differentially expressed proteins	Fold enrichment	FDR-corrected *p* value
**Primary human coronary artery endothelial cells**				
Focal adhesion (GO CC)	423	18	3.60	2.25×10^{-4}
Cell adhesion molecule binding (GO MF)	552	24	3.70	4.77×10^{-5}
Cadherin binding (GO MF)	325	16	4.20	1.13×10^{-3}
Cellular component organization or biogenesis (GO BP)	5727	100	1.50	4.84×10^{-3}
Mitochondrion (GO CC)	1671	52	2.70	1.57×10^{-8}
Extracellular vesicle (GO CC)	2123	79	3.20	4.25×10^{-18}
**Primary human internal thoracic artery endothelial cells**				
Focal adhesion (GO CC)	423	21	3.60	4.30×10^{-5}
Cell adhesion molecule binding (GO MF)	552	27	3.60	2.08×10^{-5}
Cadherin binding (GO MF)	325	15	3.40	1.55×10^{-2}
Cellular component organization or biogenesis (GO BP)	5727	119	1.50	3.02×10^{-4}
Mitochondrion (GO CC)	1671	51	2.20	6.10×10^{-6}
Extracellular vesicle (GO CC)	2123	79	2.70	1.21×10^{-13}

Supplementary Table S2. Molecular terms (selected from Gene Ontology (GO), UniProtKB Keywords, Reactome, and Kyoto Encyclopedia of Genes and Genomes (KEGG) databases) specific for human coronary artery endothelial cells (HCAEC) according to the proteomic profiling and pathway enrichment analysis. Gene Ontology database has been screened by cellular components (CC), molecular function (MF), and biological process (BP) domains.

Molecular term	Total proteins	Differentially expressed proteins	Fold enrichment	FDR-corrected *p* value
Basement membrane organisation (GO BP)	30	5	14.20	2.31×10^{-2}
Collagen-containing extracellular matrix (GO CC)	434	15	3.00	9.61×10^{-3}
Metabolism of lipids (Reactome)	736	29	3.40	1.32×10^{-5}
Fatty acid metabolic process (GO BP)	328	15	3.90	8.20×10^{-3}
Organic acid metabolic process (GO BP)	857	30	3.00	2.86×10^{-4}
Metabolism of vitamins and cofactors (Reactome)	186	11	5.00	3.26×10^{-3}
Carbohydrate metabolic process (GO BP)	475	17	3.10	2.80×10^{-2}
COPII-coated endoplasmic reticulum to Golgi transport vesicle (GO CC)	94	6	5.40	3.26×10^{-2}
Lysosome (GO CC)	749	27	3.10	2.53×10^{-5}
Peroxisome (GO CC)	146	8	4.70	1.49×10^{-2}

Supplementary Table S3. Molecular terms (selected from Gene Ontology (GO), UniProtKB Keywords, Reactome, and Kyoto Encyclopedia of Genes and Genomes (KEGG) databases) specific for human internal thoracic artery endothelial cells (HITAEC) according to the proteomic profiling and pathway enrichment analysis. Gene Ontology database has been screened by cellular components (CC), molecular function (MF), and biological process (BP) domains.

Molecular term	Total proteins	Differentially expressed proteins	Fold enrichment	FDR-corrected *p* value
Mitochondrial ribosome (GO CC)	90	16	13.00	1.36×10^{-10}
Mitochondrial small ribosomal subunit (GO CC)	32	7	16.00	4.36×10^{-5}
Mitochondrial large ribosomal subunit (GO CC)	56	9	11.70	1.39×10^{-5}
Mitochondrial gene expression (GO BP)	141	18	9.30	3.01×10^{-8}
Mitochondrial translation (GO BP)	110	17	11.20	2.27×10^{-8}
mRNA splicing via spliceosome (GO BP)	243	14	4.20	5.15×10^{-3}
RNA splicing (GO BP)	368	20	4.00	3.02×10^{-4}
Translation (GO BP)	379	24	4.60	$3.24 \times 10\text{-}^{6}$
Biosynthetic process (GO BP)	2603	65	1.80	1.32×10^{-3}
Cellular nitrogen compound metabolic process (GO BP)	3573	98	2.00	3.25×10^{-8}

Organonitrogen compound biosynthetic process (GO BP)	1333	40	2.20	2.45×10^{-3}
mRNA processing (GO BP)	451	21	3.40	1.26×10^{-3}
Macromolecule biosynthetic process (GO BP)	1487	42	2.10	5.91×10^{-3}
Elastic fibre formation (Reactome)	45	6	9.70	7.77×10^{-3}

Supplementary Table S4. Differentially expressed proteins across the cellular compartments in human coronary artery endothelial cells (HCAEC) and human internal thoracic artery endothelial cells (HITAEC), according to the Gene Ontology Cellular Component database.

Gene Ontology term	Total proteins	Differentially expressed proteins	Fold enrichment	FDR-corrected *p* value
**Primary human coronary artery endothelial cells**				
COPII-coated ER to Golgi transport vesicle	94	6	5.40	3.26×10^{-2}
Nucleosome	99	6	5.20	3.91×10^{-2}
Peroxisome	146	8	4.70	1.49×10^{-2}
Focal adhesion	423	18	3.60	2.25×10^{-4}
Extracellular vesicle	2123	79	3.20	4.25×10^{-18}
Lysosome	749	27	3.10	2.53×10^{-5}
Coated vesicle	310	11	3.00	3.83×10^{-2}
Collagen-containing extracellular matrix	434	15	3.00	9.61×10^{-3}
Mitochondrion	1671	52	2.70	1.57×10^{-8}
Endoplasmic reticulum	2045	49	2.00	8.95×10^{-5}
**Primary human internal thoracic artery endothelial cells**				
RNA polymerase I complex	13	3	16.80	2.79×10^{-2}
Mitochondrial small ribosomal subunit	32	7	16.00	4.36×10^{-5}
Mitochondrial ribosome	90	16	13.00	1.36×10^{-10}
Mitochondrial large ribosomal subunit	56	9	11.70	1.39×10^{-5}
Ribosomal subunit	189	18	6.90	4.60×10^{-8}
Ribosome	118	18	5.80	6.35×10^{-7}
Focal adhesion	423	21	3.60	4.30×10^{-5}
Extracellular vesicle	2123	79	2.70	1.21×10^{-13}
Cell-cell junction	508	17	2.40	2.19×10^{-2}

Mitochondrion	1671	51	2.20	6.10×10^{-6}

Supplementary Table S5. Differentially expressed proteins across the cellular compartments in human coronary artery endothelial cells (HCAEC) and human internal thoracic artery endothelial cells (HITAEC), according to the UniProtKB Keywords database.

**Pathway**	**Total proteins**	**Percent from differentially expressed proteins**	**Fold enrichment**	**FDR-corrected *p* value**
**Primary human coronary artery endothelial cells**				
Nucleosome core	16	6.2	12.90	5.50×10^{-11}
Mitochondrion outer membrane	8	3.1	4.00	3.10×10^{-2}
Chromosome	25	9.8	3.20	2.60×10^{-5}
Lysosome	13	5.1	2.50	3.40×10^{-2}
Mitochondrion	39	15.2	2.20	7.90×10^{-5}
Endoplasmic reticulum	38	14.8	2.10	1.40×10^{-4}
**Primary human internal thoracic artery endothelial cells**				
Spliceosome	10	3.5	4.70	5.60×10^{-3}
Mitochondrion	42	14.5	2.20	1.30×10^{-4}

Supplementary Table S6. Molecular functions of proteins which are differentially expressed human coronary artery endothelial cells (HCAEC) and human internal thoracic artery endothelial cells (HITAEC), according to the Gene Ontology Molecular Function database.

**Gene Ontology term**	**Total proteins**	**Differentially expressed proteins**	**Fold enrichment**	**FDR-corrected *p* value**
**Primary human coronary artery endothelial cells**				
Cuprous ion binding	8	3	32.00	4.88×10^{-2}
Structural constituent of chromatin	66	6	7.80	4.03×10^{-2}
Cadherin binding	325	16	4.20	1.13×10^{-3}
Oxidoreductase activity	741	33	3.80	2.55×10^{-7}
Cell adhesion molecule binding	552	24	3.70	4.77×10^{-5}

**Primary human internal thoracic artery endothelial cells**				
Insulin-like growth factor binding	20	5	18.20	6.14×10^{-3}
Ephrin receptor binding	30	5	12.20	2.29×10^{-2}
Extracellular matrix binding	56	7	9.10	6.47×10^{-3}
Cell adhesion molecule binding	552	27	3.60	2.08×10^{-5}
Cadherin binding	325	15	3.40	1.55×10^{-2}

Supplementary Table S7. Molecular functions of proteins which are differentially expressed human coronary artery endothelial cells (HCAEC) and human internal thoracic artery endothelial cells (HITAEC), according to the UniProtKB Keywords database.

Pathway	Total proteins	Percent from differentially expressed proteins	Fold enrichment	FDR-corrected *p* value
**Primary human coronary artery endothelial cells**				
Isomerase	12	4.7	5.20	5.90×10^{-4}
Oxidoreductase	25	9.8	2.70	5.90×10^{-4}
**Primary human internal thoracic artery endothelial cells**				
Growth factor binding	4	1.4	14.30	3.90×10^{-2}
Ribosomal protein	16	5.5	5.90	4.40×10^{-6}
Ribonucleoprotein	17	5.9	4.00	1.60×10^{-4}
Chaperone	12	4.2	3.80	5.90×10^{-3}

Supplementary Table S8. Biological processes which involve proteins that are differentially expressed in human coronary artery endothelial cells (HCAEC) and human internal thoracic artery endothelial cells (HITAEC), according to the Gene Ontology Biological Process database.

Gene Ontology term	Total proteins	Differentially expressed proteins	Fold enrichment	FDR-corrected *p* value
**Primary human coronary artery endothelial cells**				
Basement membrane organization	30	5	14.20	2.31×10^{-2}

Fatty acid catabolic process	85	8	8.00	8.16×10^{-3}
Electron transport chain	167	10	5.10	2.08×10^{-2}
Fatty acid metabolic process	328	15	3.90	8.20×10^{-3}
Generation of precursor metabolites and energy	406	18	3.80	2.23×10^{-3}
Mitochondrion organization	445	16	3.10	4.04×10^{-2}
Carbohydrate metabolic process	475	17	3.10	2.80×10^{-2}
Organic acid metabolic process	857	30	3.00	2.86×10^{-4}
Membrane organization	851	28	2.80	1.55×10^{-3}
Endomembrane system organization	554	18	2.80	4.47×10^{-2}
Lipid biosynthetic process	604	19	2.70	4.46×10^{-2}
Lipid metabolic process	1234	37	2.60	3.18×10^{-4}
Organophosphate metabolic process	931	25	2.30	4.73×10^{-2}
Cellular component biogenesis	2633	53	1.70	3.80×10^{-2}
Cellular component organization or biogenesis	5727	100	1.50	4.84×10^{-3}
**Primary human internal thoracic artery endothelial cells**				
Mitochondrial translation	110	17	11.20	2.27×10^{-8}
Mitochondrial gene expression	141	18	9.30	3.01×10^{-8}
Regulation of cell shape	155	11	5.20	6.71×10^{-3}
Translation	379	24	4.60	3.24×10^{-6}
mRNA splicing, via spliceosome	243	14	4.20	5.15×10^{-3}
RNA splicing	368	20	4.00	3.02×10^{-4}
mRNA processing	451	21	3.40	1.26×10^{-3}
Positive regulation of cell motility	558	22	2.90	6.00×10^{-3}
Cellular nitrogen compound biosynthetic process	1588	54	2.50	2.25×10^{-6}
RNA processing	868	29	2.40	6.79×10^{-3}
Organonitrogen compound biosynthetic process	1333	40	2.20	2.45×10^{-3}
Macromolecule biosynthetic process	1487	42	2.10	5.91×10^{-3}
Cellular nitrogen compound metabolic process	3573	98	2.00	3.25×10^{-8}
Biosynthetic process	2603	65	1.80	1.32×10^{-3}
Cellular component organization or biogenesis	5727	119	1.50	3.02×10^{-4}

Supplementary Table S9. Biological processes which involve proteins that are differentially expressed in human coronary artery endothelial cells (HCAEC) and human internal thoracic artery endothelial cells (HITAEC), according to the Reactome database.

**Reactome pathway**	**Total proteins**	**Differentially expressed proteins**	**Fold enrichment**	**FDR-corrected *p* value**
**Primary human coronary artery endothelial cells**				
Cholesterol biosynthesis	24	4	14.20	3.26×10^{-2}
RHOJ GTPase cycle	54	8	12.70	2.34×10^{-4}
Transcriptional regulation by small RNAs	73	6	7.00	3.36×10^{-2}
Metabolism of water-soluble vitamins and cofactors	120	8	5.70	1.63×10^{-2}
Fatty acid metabolism	174	11	5.40	1.94×10^{-3}
Metabolism of vitamins and cofactors	186	11	5.00	3.26×10^{-3}
RHO GTPase Effectors	288	12	3.60	2.46×10^{-2}
Metabolism of lipids	736	29	3.40	1.32×10^{-5}
Signaling by Rho GTPases	670	24	3.10	3.98×10^{-4}
RHO GTPase cycle	448	16	3.00	1.50×10^{-2}
**Primary human internal thoracic artery endothelial cells**				
PECAM1 interactions	11	3	19.90	3.74×10^{-2}
Mitochondrial translation	94	16	12.40	1.12×10^{-9}
Elastic fibre formation	45	6	9.70	7.77×10^{-3}
trans-Golgi Network Vesicle Budding	72	9	9.10	3.06×10^{-4}
Integrin cell surface interactions	84	9	7.80	7.84×10^{-4}
Post-translational protein phosphorylation	107	11	7.50	1.50×10^{-4}
ECM proteoglycans	76	7	6.70	1.43×10^{-2}
mRNA Splicing	188	14	5.40	1.57×10^{-4}
Nucleotide Excision Repair	110	8	5.30	1.75×10^{-2}
Cell surface interactions at the vascular wall	197	10	3.70	3.51×10^{-2}

Supplementary Table S10. Biological processes which involve proteins that are differentially expressed in human coronary artery endothelial cells (HCAEC) and human internal thoracic artery endothelial cells (HITAEC), according to the Kyoto Encyclopedia of Genes and Genomes (KEGG) database.

Pathway	Total proteins	Percent from differentially expressed proteins	Fold enrichment	FDR-corrected *p* value
**Primary human coronary artery endothelial cells**				
Lipid metabolism	23	9.0	2.60	4.70×10^{-3}
**Primary human internal thoracic artery endothelial cells**				
mRNA splicing	17	5.9	3.60	1.30×10^{-3}
mRNA processing	19	6.6	3.10	1.30×10^{-3}
Cell adhesion	18	6.2	2.30	4.00×10^{-2}

Supplementary Table S11. Biochemical pathways which involve proteins that are differentially expressed in human coronary artery endothelial cells (HCAEC) and human internal thoracic artery endothelial cells (HITAEC), according to the Kyoto Encyclopedia of Genes and Genomes (KEGG) database.

Pathway	Total proteins	Percent from differentially expressed proteins	Fold enrichment	FDR-corrected *p* value
**Primary human coronary artery endothelial cells**				
Neutrophil extracellular trap formation	19	7.4	5.10	6.60×10^{-6}
Metabolic pathways	51	19.9	1.70	4.10×10^{-3}
**Primary human internal thoracic artery endothelial cells**				
Spliceosome	11	3.8	4.00	5.10×10^{-2}

TRANSCRIPTOMIC PROFILING UNDER LAMINAR FLOW

Supplementary Table S12. Molecular terms (selected from Gene Ontology (GO), UniProtKB Keywords, Reactome, and Kyoto Encyclopedia of Genes and Genomes (KEGG) databases) common for human coronary artery endothelial cells (HCAEC) and human internal thoracic artery endothelial cells (HITAEC) cultured under laminar flow according to the RNA sequencing and pathway enrichment analysis. Gene Ontology database has been screened by cellular components (CC), molecular function (MF), and biological process (BP) domains.

Molecular term	Total genes	Differentially expressed genes	Fold enrichment	FDR-corrected *p* value
**Primary human coronary artery endothelial cells**				
Collagen-containing extracellular matrix (GO CC)	434	43	2.40	2.99×10^{-5}
Extracellular matrix (GO CC)	575	54	2.30	7.38×10^{-6}
Cell-matrix adhesion (GO BP)	135	15	2.70	4.38×10^{-2}
Glycosaminoglycan binding (GO MF)	242	28	2.80	1.07×10^{-3}
Calcium signaling pathway (KEGG)	25	3	2.40	3.5×10^{-3}
Cell junction (UniProtKB Keywords)	82	9	2.30	1.7×10^{-10}
Secreted (UniProtKB Keywords)	123	13	1.40	4.9×10^{-4}
DNA repair (GO BP)	508	7	0.30	3.98×10^{-2}
RNA processing (GO BP)	868	9	0.25	4.30×10^{-5}
Protein ubiquitination (GO BP)	684	11	0.40	2.66×10^{-2}
Macroautophagy (GO BP)	189	0	0.01	4.64×10^{-2}
**Primary human internal thoracic artery endothelial cells**				
Collagen-containing extracellular matrix (GO CC)	434	43	2.80	1.35×10^{-6}
Extracellular matrix (GO CC)	575	63	3.10	1.72×10^{-11}
Cell-substrate adhesion (GO BP)	194	19	2.80	1.01×10^{-2}
Glycosaminoglycan binding (GO MF)	242	25	2.90	3.77×10^{-3}
Calcium signaling pathway (KEGG)	22	3	2.40	2.3×10^{-2}
Cell junction (UniProtKB Keywords)	53	7	1.70	2.7×10^{-3}
Secreted (UniProtKB Keywords)	137	17	1.80	4.0×10^{-10}
DNA repair (GO BP)	508	3	0.17	3.83×10^{-3}
RNA processing (GO BP)	868	3	0.10	2.92×10^{-7}
Ubiquitin-dependent protein catabolic process (GO BP)	575	6	0.30	2.55×10^{-2}

Autophagy (GO BP)	303	1	0.10	3.39×10^{-2}

Supplementary Table S13. Molecular terms (selected from Gene Ontology (GO), UniProtKB Keywords, Reactome, and Kyoto Encyclopedia of Genes and Genomes (KEGG) databases) specific for human coronary artery endothelial cells (HCAEC) cultured under laminar flow according to the RNA sequencing and pathway enrichment analysis. Gene Ontology database has been screened by cellular components (CC), molecular function (MF), and biological process (BP) domains.

Molecular term	Total genes	Differentially expressed genes	Fold enrichment	FDR-corrected *p* value
Collagen trimer (GO CC)	88	13	3.60	4.77×10^{-3}
Collagen-containing extracellular matrix (GO CC)	434	43	2.40	2.99×10^{-5}
Collagen biosynthesis and modifying enzymes (Reactome)	67	12	4.30	1.41×10^{-2}
Collagen formation (Reactome)	89	13	3.50	3.49×10^{-2}
Collagen binding (GO MF)	70	15	5.20	6.19×10^{-4}
Basal plasma membrane (GO CC)	256	28	2.60	3.84×10^{-4}
Cell-cell junction (GO CC)	508	43	2.00	9.83×10^{-4}
Proteoglycan binding (GO MF)	37	10	6.50	2.29×10^{-3}
Integrin binding (GO MF)	159	23	3.50	7.84×10^{-4}
Elastic fiber assembly (GO BP)	12	5	10.10	2.30×10^{-2}

Supplementary Table S14. Molecular terms (selected from Gene Ontology (GO), UniProtKB Keywords, Reactome, and Kyoto Encyclopedia of Genes and Genomes (KEGG) databases) specific for human internal thoracic artery endothelial cells (HITAEC) cultured under laminar flow according to the RNA sequencing and pathway enrichment analysis. Gene Ontology database has been screened by cellular components (CC), molecular function (MF), and biological process (BP) domains.

Molecular term	Total genes	Differentially expressed genes	Fold enrichment	FDR-corrected *p* value
Cortical cytoskeleton (GO CC)	111	13	3.30	1.30×10^{-2}
Actin cytoskeleton (GO CC)	510	34	1.90	2.49×10^{-2}
Actin cytoskeleton organization (GO BP)	549	40	2.10	4.26×10^{-3}

Glycosaminoglycan biosynthetic process (GO BP)	70	10	4.00	2.26×10^{-2}
Glycosaminoglycan metabolism (Reactome)	123	16	3.70	1.07×10^{-2}
Extracellular vesicle (GO CC)	2123	107	1.40	1.08×10^{-2}
Positive regulation of chemokine production (GO BP)	71	10	4.00	2.50×10^{-2}
Positive regulation of CXCL2 production (GO BP)	10	4	11.30	4.92×10^{-2}
Positive regulation of interleukin-6 production (GO BP)	98	13	3.80	8.50×10^{-3}
Positive regulation of cytokine production (GO BP)	481	37	2.20	2.74×10^{-3}

Supplementary Table S15. Differentially expressed genes across the cellular compartments in human coronary artery endothelial cells (HCAEC) and human internal thoracic artery endothelial cells (HITAEC) cultured under laminar flow, according to the Gene Ontology Cellular Component database.

**Gene Ontology term**	**Total genes**	**Differentially expressed genes**	**Fold enrichment**	**FDR-corrected *p* value**
**Primary human coronary artery endothelial cells**				
Collagen trimer	88	13	3.60	4.77×10^{-3}
Basement membrane	101	14	3.40	4.84×10^{-3}
Basal plasma membrane	256	28	2.60	3.84×10^{-4}
Collagen-containing extracellular matrix	434	43	2.40	2.99×10^{-5}
Extracellular matrix	575	54	2.30	7.38×10^{-6}
Cell-cell junction	508	43	2.00	9.83×10^{-4}
Microtubule cytoskeleton	1374	35	0.60	4.74×10^{-2}
Mitochondrion	1671	36	0.50	5.04×10^{-4}
Mitochondrial inner membrane	497	6	0.30	1.08×10^{-2}
Ribonucleoprotein complex	714	9	0.30	7.98×10^{-4}
**Primary human internal thoracic artery endothelial cells**				
Cortical cytoskeleton	111	13	3.30	1.30×10^{-2}
Golgi lumen	104	12	3.30	2.24×10^{-2}
Lysosomal lumen	98	11	3.20	4.00×10^{-2}
Sarcomere	215	18	2.40	4.45×10^{-2}

Collagen-containing extracellular matrix	434	43	2.80	1.35×10^{-6}
Extracellular matrix	575	63	3.10	1.72×10^{-11}
Transport vesicle	429	29	1.90	4.77×10^{-2}
Actin cytoskeleton	510	34	1.90	2.49×10^{-2}
Extracellular vesicle	2123	107	1.40	1.08×10^{-2}
Ribonucleoprotein complex	714	2	0.08	1.36×10^{-6}

Supplementary Table S16. Differentially expressed genes across the cellular compartments in human coronary artery endothelial cells (HCAEC) and human internal thoracic artery endothelial cells (HITAEC) cultured under laminar flow, according to the UniProtKB Keywords database.

**Pathway**	**Total genes**	**Percent from differentially expressed genes**	**Fold enrichment**	**FDR-corrected *p* value**
**Primary human coronary artery endothelial cells**				
Extracellular matrix	28	2.9	2.50	1.90×10^{-4}
Cell junction	82	8.5	2.30	1.70×10^{-10}
Cell membrane	233	24.2	1.50	1.30×10^{-9}
Secreted	123	12.8	1.40	4.90×10^{-4}
Membrane	411	42.7	1.20	3.10×10^{-7}
**Primary human internal thoracic artery endothelial cells**				
Extracellular matrix	32	4.0	3.20	4.20×10^{-7}
Secreted	137	17.1	1.80	4.00×10^{-10}
Cell junction	53	6.6	1.70	2.70×10^{-3}
Cell projection	65	8.1	1.60	3.00×10^{-3}
Cell membrane	191	23.8	1.40	1.10×10^{-5}

Supplementary Table S17. Molecular functions of genes which are differentially expressed human coronary artery endothelial cells (HCAEC) and human internal thoracic artery endothelial cells (HITAEC) cultured under laminar flow, according to the Gene Ontology Molecular Function database.

Gene Ontology term	Total genes	Differentially expressed genes	Fold enrichment	FDR-corrected *p* value
**Primary human coronary artery endothelial cells**				
Proteoglycan binding	37	10	6.50	2.29×10^{-3}
Collagen binding	70	15	5.20	6.19×10^{-4}
Integrin binding	159	23	3.50	7.84×10^{-4}
Glycosaminoglycan binding	242	28	2.80	1.07×10^{-3}
Cell adhesion molecule binding	552	56	2.50	2.43×10^{-5}
**Primary human internal thoracic artery endothelial cells**				
Chemorepellent activity	29	9	8.80	3.27×10^{-3}
Receptor ligand activity	497	39	2.20	5.15×10^{-3}
Glycosaminoglycan binding	242	25	2.90	3.77×10^{-3}
Calcium ion binding	753	57	2.20	1.91×10^{-4}
RNA binding	1666	17	0.30	8.06×10^{-7}

Supplementary Table S18. Molecular functions of genes which are differentially expressed human coronary artery endothelial cells (HCAEC) and human internal thoracic artery endothelial cells (HITAEC) cultured under laminar flow, according to the UniProtKB Keywords database.

Pathway	Total genes	Percent from differentially expressed genes	Fold enrichment	FDR-corrected *p* value
**Primary human coronary artery endothelial cells**				
Sodium channel	9	0.9	7.50	2.30×10^{-4}
Heparin-binding	14	1.5	3.60	1.30×10^{-3}
Calcium channel	10	1.0	3.60	1.70×10^{-2}
Voltage-gated channel	22	2.3	3.50	1.60×10^{-5}
Ion channel	39	4.0	2.60	2.90×10^{-6}
**Primary human internal thoracic artery endothelial cells**				
Integrin	8	1.0	4.70	2.60×10^{-2}

Growth factor	14	1.7	2.90	2.60×10^{-2}
Actin-binding	22	2.7	2.10	3.10×10^{-2}

Supplementary Table S19. Biological processes which involve genes that are differentially expressed in human coronary artery endothelial cells (HCAEC) and human internal thoracic artery endothelial cells (HITAEC) cultured under laminar flow, according to the Gene Ontology Biological Process database.

**Gene Ontology term**	**Total genes**	**Differentially expressed genes**	**Fold enrichment**	**FDR-corrected *p* value**
**Primary human coronary artery endothelial cells**				
Elastic fiber assembly	12	5	10.10	2.30×10^{-2}
Heterophilic cell-cell adhesion via plasma membrane cell adhesion molecules	52	13	6.10	2.17×10^{-4}
Artery morphogenesis	67	10	3.60	4.29×10^{-2}
Homophilic cell adhesion via plasma membrane adhesion molecules	170	22	3.10	1.06×10^{-3}
Cell-cell adhesion	543	69	3.10	6.16×10^{-12}
Cell adhesion	969	107	2.70	3.64×10^{-15}
Cell-matrix adhesion	135	15	2.70	4.38×10^{-2}
Extracellular matrix organization	280	28	2.40	4.19×10^{-3}
Nucleic acid metabolic process	2276	46	0.50	6.22×10^{-6}
DNA metabolic process	794	15	0.50	4.16×10^{-2}
Protein ubiquitination	684	11	0.40	2.66×10^{-2}
DNA repair	508	7	0.30	3.98×10^{-2}
RNA processing	868	9	0.25	4.30×10^{-5}
Ribonucleoprotein complex biogenesis	449	3	0.16	2.56×10^{-3}
Macroautophagy	189	0	0.01	4.64×10^{-2}
**Primary human internal thoracic artery endothelial cells**				
Positive regulation of chemokine (C-X-C motif) ligand 2 production	10	4	11.30	4.92×10^{-2}
cGMP-mediated signaling	28	7	7.10	1.10×10^{-2}
Collagen fibril organization	60	9	4.30	3.06×10^{-2}
Glycosaminoglycan biosynthetic process	70	10	4.00	2.26×10^{-2}

Response to cAMP	93	13	4.00	5.91×10^{-3}
Positive regulation of chemokine production	71	10	4.00	2.50×10^{-2}
Positive regulation of interleukin-6 production	98	13	3.80	8.50×10^{-3}
Positive regulation of phagocytosis	76	10	3.70	3.64×10^{-2}
Extracellular matrix organization	280	31	3.10	3.30×10^{-5}
Cell-substrate adhesion	194	19	2.80	1.01×10^{-2}
Positive regulation of leukocyte migration	152	15	2.80	3.37×10^{-2}
Vasculature development	532	46	2.40	3.83×10^{-5}
Positive regulation of cell-cell adhesion	324	27	2.40	7.19×10^{-3}
Positive regulation of cytokine production	481	37	2.20	2.74×10^{-3}
Actin cytoskeleton organization	549	40	2.10	4.26×10^{-3}
Ubiquitin-dependent protein catabolic process	575	6	0.30	2.55×10^{-2}
DNA repair	508	3	0.17	3.83×10^{-3}
Translation	379	2	0.15	1.90×10^{-2}
Ribonucleoprotein complex biogenesis	449	2	0.13	3.83×10^{-3}
RNA processing	868	3	0.10	2.92×10^{-7}
Autophagy	303	1	0.10	3.39×10^{-2}

Supplementary Table S20. Biological processes which involve genes that are differentially expressed in human coronary artery endothelial cells (HCAEC) and human internal thoracic artery endothelial cells (HITAEC) cultured under laminar flow, according to the Reactome database.

**Reactome pathway**	**Total genes**	**Differentially expressed genes**	**Fold enrichment**	**FDR-corrected *p* value**
**Primary human coronary artery endothelial cells**				
Cell-cell junction organization	64	12	4.50	1.16×10^{-2}
Collagen biosynthesis and modifying enzymes	67	12	4.30	1.41×10^{-2}
Collagen formation	89	13	3.50	3.49×10^{-2}
**Primary human internal thoracic artery endothelial cells**				
Glycosaminoglycan metabolism	123	16	3.70	1.07×10^{-2}

Supplementary Table S21. Biological processes which involve genes that are differentially expressed in human coronary artery endothelial cells (HCAEC) and human internal thoracic artery endothelial cells (HITAEC) cultured under laminar flow, according to the Kyoto Encyclopedia of Genes and Genomes (KEGG) database.

**Pathway**	**Total genes**	**Percent from differentially expressed genes**	**Fold enrichment**	**FDR-corrected *p* value**
**Primary human coronary artery endothelial cells**				
Cell adhesion	69	7.2	3.70	3.00×10^{-19}
Calcium transport	14	1.5	3.60	3.30×10^{-3}
Sodium transport	14	1.5	3.00	1.60×10^{-2}
Ion transport	57	5.9	2.30	6.10×10^{-7}
Differentiation	52	5.4	1.80	1.90×10^{-3}
**Primary human internal thoracic artery endothelial cells**				
Cell adhesion	36	4.5	2.30	4.00×10^{-4}

Supplementary Table S22. Biochemical pathways which involve genes that are differentially expressed in human coronary artery endothelial cells (HCAEC) and human internal thoracic artery endothelial cells (HITAEC) cultured under laminar flow, according to the Kyoto Encyclopedia of Genes and Genomes (KEGG) database.

**Pathway**	**Total genes**	**Percent from differentially expressed genes**	**Fold enrichment**	**FDR-corrected *p* value**
**Primary human coronary artery endothelial cells**				
Cell adhesion molecules	24	2.5	3.60	2.30×10^{-5}
Protein digestion and absorption	13	1.3	3.00	4.50×10^{-2}
cAMP signaling pathway	24	2.5	2.60	3.20×10^{-3}
Calcium signaling pathway	25	2.6	2.40	3.50×10^{-3}
**Primary human internal thoracic artery endothelial cells**				
ECM-receptor interaction	13	1.6	3.90	2.30×10^{-2}

Phospholipase D signaling pathway	16	2.0	2.90	2.90×10^{-2}
Focal adhesion	20	2.5	2.60	2.30×10^{-2}
Calcium signaling pathway	22	2.7	2.40	2.30×10^{-2}

TRANSCRIPTOMIC PROFILING AT STATIC CELL CULTURE CONDITIONS

Supplementary Table S23. Molecular terms (selected from Gene Ontology (GO), UniProtKB Keywords, Reactome, and Kyoto Encyclopedia of Genes and Genomes (KEGG) databases) common for human coronary artery endothelial cells (HCAEC) and human internal thoracic artery endothelial cells (HITAEC) cultured under static conditions according to the RNA sequencing and pathway enrichment analysis. Gene Ontology database has been screened by cellular components (CC), molecular function (MF), and biological process (BP) domains.

**Molecular term**	**Total genes**	**Differentially expressed genes**	**Fold enrichment**	**FDR-corrected *p* value**
**Primary human coronary artery endothelial cells**				
Collagen-containing extracellular matrix (GO CC)	434	32	2.40	2.51×10^{-3}
Extracellular matrix (GO CC)	575	35	2.00	3.30×10^{-2}
Cell junction assembly (GO BP)	276	21	2.50	1.59×10^{-2}
Cell junction (UniProtKB Keywords)	50	7	1.90	6.1×10^{-4}
Extracellular vesicle (GO CC)	2123	92	1.40	4.87×10^{-2}
Secreted (UniProtKB Keywords)	91	13	1.40	3.8×10^{-3}
**Primary human internal thoracic artery endothelial cells**				
Collagen-containing extracellular matrix (GO CC)	434	32	1.90	3.14×10^{-2}
Extracellular matrix (GO CC)	575	42	1.90	1.13×10^{-2}
Cell-matrix adhesion (GO BP)	135	15	2.80	4.51×10^{-2}
Cell junction (UniProtKB Keywords)	56	6	1.60	5.2×10^{-3}
Secretory vesicle (GO CC)	1051	64	1.50	2.50×10^{-2}
Secreted (UniProtKB Keywords)	127	14	1.50	1.3×10^{-4}

Supplementary Table S24. Molecular terms (selected from Gene Ontology (GO), UniProtKB Keywords, Reactome, and Kyoto Encyclopedia of Genes and Genomes (KEGG) databases) specific for human coronary artery endothelial cells (HCAEC) cultured under

static conditions according to the RNA sequencing and pathway enrichment analysis. Gene Ontology database has been screened by cellular components (CC), molecular function (MF), and biological process (BP) domains.

Molecular term	Total genes	Differentially expressed genes	Fold enrichment	FDR-corrected *p* value
Artery development (GO BP)	95	11	3.90	2.19×10^{-2}
Vasculature development (GO BP)	532	41	2.60	7.67×10^{-5}
Cellular nitrogen compound biosynthetic process (GO BP)	1588	26	0.50	4.30×10^{-2}
Proteolysis involved in protein catabolic process (GO BP)	647	6	0.30	4.92×10^{-2}

Supplementary Table S25. Molecular terms (selected from Gene Ontology (GO), UniProtKB Keywords, Reactome, and Kyoto Encyclopedia of Genes and Genomes (KEGG) databases) specific for human internal thoracic artery endothelial cells (HITAEC) cultured under static conditions according to the RNA sequencing and pathway enrichment analysis. Gene Ontology database has been screened by cellular components (CC), molecular function (MF), and biological process (BP) domains.

Molecular term	Total genes	Differentially expressed genes	Fold enrichment	FDR-corrected *p* value
Early endosome (GO CC)	415	32	2.00	1.85×10^{-2}
Endosome (GO CC)	1036	69	1.70	3.38×10^{-3}
Lysosome (GO CC)	749	53	1.80	5.70×10^{-3}
Inflammatory response (GO BP)	540	40	1.90	2.32×10^{-2}
Macromolecule biosynthetic process (GO BP)	1487	31	0.50	1.11×10^{-2}
DNA repair (GO BP)	508	5	0.25	1.64×10^{-2}
RNA processing (GO BP)	868	4	0.12	8.31×10^{-7}
RNA splicing (GO BP)	368	1	0.07	4.40×10^{-3}
Ribosome biogenesis (GO BP)	303	1	0.08	2.01×10^{-2}
Translation (GO BP)	379	2	0.13	1.29×10^{-2}

Supplementary Table S26. Differentially expressed genes across the cellular compartments in human coronary artery endothelial cells (HCAEC) and human internal thoracic artery endothelial cells (HITAEC) cultured under static conditions, according to the Gene Ontology Cellular Component database.

Gene Ontology term	Total genes	Differentially expressed genes	Fold enrichment	FDR-corrected *p* value
**Primary human coronary artery endothelial cells**				
Collagen-containing extracellular matrix	434	32	2.40	2.51×10^{-3}
Cell-cell junction	508	32	2.10	2.96×10^{-2}
Extracellular matrix	575	35	2.00	3.30×10^{-2}
Extracellular vesicle	2123	92	1.40	4.87×10^{-2}
**Primary human internal thoracic artery endothelial cells**				
Laminin complex	12	5	10.60	1.18×10^{-2}
Cortical cytoskeleton	111	14	3.20	1.12×10^{-2}
Phagocytic vesicle	140	14	2.50	4.91×10^{-2}
Early endosome	415	32	2.00	1.85×10^{-2}
Collagen-containing extracellular matrix	434	32	1.90	3.14×10^{-2}
Extracellular matrix	575	42	1.90	1.13×10^{-2}
Lysosome	749	53	1.80	5.70×10^{-3}
Endosome	1036	69	1.70	3.38×10^{-3}
Secretory vesicle	1051	64	1.50	2.50×10^{-2}
Anchoring junction	1336	76	1.50	4.23×10^{-2}

Supplementary Table S27. Differentially expressed genes across the cellular compartments in human coronary artery endothelial cells (HCAEC) and human internal thoracic artery endothelial cells (HITAEC) cultured under static conditions, according to the UniProtKB Keywords database.

Pathway	Total genes	Percent from differentially expressed genes	Fold enrichment	FDR-corrected *p* value

**Primary human coronary artery endothelial cells**				
Extracellular matrix	22	3.2	2.70	1.60×10^{-3}
Cell junction	50	7.2	1.90	6.10×10^{-4}
Secreted	91	13.1	1.40	3.80×10^{-3}
Cell membrane	149	21.5	1.30	3.80×10^{-3}
**Primary human internal thoracic artery endothelial cells**				
Lysosome	27	3.0	1.80	3.00×10^{-2}
Cell junction	56	6.3	1.60	5.20×10^{-3}
Endosome	41	4.6	1.60	2.80×10^{-2}
Secreted	127	14.2	1.50	1.30×10^{-4}
Cell membrane	197	22.1	1.30	1.10×10^{-3}

Supplementary Table S28. Molecular functions of genes which are differentially expressed human coronary artery endothelial cells (HCAEC) and human internal thoracic artery endothelial cells (HITAEC) cultured under static conditions, according to the Gene Ontology Molecular Function database.

Gene Ontology term	Total genes	Differentially expressed genes	Fold enrichment	FDR-corrected *p* value
**Primary human coronary artery endothelial cells**				
Actin filament binding	222	21	3.10	2.50×10^{-2}
Calcium ion binding	735	45	2.00	2.82×10^{-2}
Signaling receptor binding	1602	82	1.70	2.46×10^{-2}
**Primary human internal thoracic artery endothelial cells**				
Carbohydrate derivative binding	2287	131	1.50	1.26×10^{-2}
RNA binding	1666	29	0.44	4.77×10^{-4}

Supplementary Table S29. Molecular functions of genes which are differentially expressed human coronary artery endothelial cells (HCAEC) and human internal thoracic artery endothelial cells (HITAEC) cultured under static conditions, according to the UniProtKB Keywords database.

Pathway	Total genes	Percent from differentially expressed genes	Fold enrichment	FDR-corrected *p* value
**Primary human coronary artery endothelial cells**				
Growth factor	14	2.0	3.50	4.30×10^{-3}
**Primary human internal thoracic artery endothelial cells**				
None				

Supplementary Table S30. Biological processes which involve genes that are differentially expressed in human coronary artery endothelial cells (HCAEC) and human internal thoracic artery endothelial cells (HITAEC) cultured under static conditions, according to the Gene Ontology Biological Process database.

Gene Ontology term	Total genes	Differentially expressed genes	Fold enrichment	FDR-corrected *p* value
**Primary human coronary artery endothelial cells**				
Regulation of transforming growth factor beta production	39	7	6.00	2.60×10^{-2}
Endothelial cell migration	73	9	4.10	4.17×10^{-2}
Artery development	95	11	3.90	2.19×10^{-2}
Cell-cell junction organization	176	18	3.40	2.80×10^{-3}
Negative regulation of cell motility	303	25	2.70	2.45×10^{-3}
Vasculature development	532	41	2.60	7.67×10^{-5}
Cell junction assembly	276	21	2.50	1.59×10^{-2}
Regulation of Wnt signaling pathway	332	24	2.40	1.45×10^{-2}
Cell junction organization	498	35	2.30	1.88×10^{-3}
Actin cytoskeleton organization	549	36	2.20	4.07×10^{-3}
Cell-cell adhesion	543	33	2.00	1.90×10^{-2}
Positive regulation of cell differentiation	884	52	2.00	1.84×10^{-3}
Cellular nitrogen compound biosynthetic process	1588	26	0.50	4.30×10^{-2}
Proteolysis involved in protein catabolic process	647	6	0.30	4.92×10^{-2}
Ubiquitin-dependent protein catabolic process	575	4	0.23	2.68×10^{-2}

**Primary human internal thoracic artery endothelial cells**				
Blood vessel diameter maintenance	144	16	2.80	3.24×10^{-2}
Cell-matrix adhesion	135	15	2.80	4.51×10^{-2}
Positive regulation of cell-cell adhesion	324	34	2.70	3.87×10^{-4}
Positive regulation of cell adhesion	488	49	2.60	2.37×10^{-5}
Positive regulation of I-kappaB kinase/NF-kappaB signaling	190	19	2.50	3.21×10^{-2}
Positive regulation of cytokine production	481	37	2.00	2.31×10^{-2}
Positive regulation of cell motility	558	44	2.00	5.32×10^{-3}
Inflammatory response	540	40	1.90	2.32×10^{-2}
Cell adhesion	969	66	1.70	5.97×10^{-3}
Macromolecule biosynthetic process	1487	31	0.50	1.11×10^{-2}
DNA repair	508	5	0.25	1.64×10^{-2}
Translation	379	2	0.13	1.29×10^{-2}
RNA processing	868	4	0.12	8.31×10^{-7}
Ribosome biogenesis	303	1	0.08	2.01×10^{-2}
RNA splicing	368	1	0.07	4.40×10^{-3}

Supplementary Table S31. Biological processes which involve genes that are differentially expressed in human coronary artery endothelial cells (HCAEC) and human internal thoracic artery endothelial cells (HITAEC) cultured under static conditions, according to the Reactome database.

**Reactome pathway**	**Total genes**	**Differentially expressed genes**	**Fold enrichment**	**FDR-corrected *p* value**
**Primary human coronary artery endothelial cells**				
None				
**Primary human internal thoracic artery endothelial cells**				
None				

Supplementary Table S32. Biological processes which involve genes that are differentially expressed in human coronary artery endothelial cells (HCAEC) and human internal thoracic artery endothelial cells (HITAEC) cultured under static conditions, according to the Kyoto Encyclopedia of Genes and Genomes (KEGG) database.

Pathway	Total genes	Percent from differentially expressed genes	Fold enrichment	FDR-corrected *p* value
**Primary human coronary artery endothelial cells**				
Cell adhesion	36	5.2	2.80	8.00×10^{-6}
**Primary human internal thoracic artery endothelial cells**				
Innate immunity	37	4.1	2.50	6.70×10^{-5}
Cell adhesion	36	4.0	1.90	8.70×10^{-3}
Immunity	56	6.3	1.60	8.70×10^{-3}

Supplementary Table S33. Biochemical pathways which involve genes that are differentially expressed in human coronary artery endothelial cells (HCAEC) and human internal thoracic artery endothelial cells (HITAEC) cultured under static conditions, according to the Kyoto Encyclopedia of Genes and Genomes (KEGG) database.

Pathway	Total genes	Percent from differentially expressed genes	Fold enrichment	FDR-corrected *p* value
**Primary human coronary artery endothelial cells**				
None				
**Primary human internal thoracic artery endothelial cells**				
Cell adhesion molecules	21	2.4	2.90	1.10×10^{-2}
Phagosome	20	2.2	2.80	1.20×10^{-2}
Calcium signaling pathway	24	2.7	2.10	4.70×10^{-2}

BIOINFORMATIC ANALYSIS OF HCAEC-HITAEC INTERACTOME

Supplementary Table S34. Molecular categories enriched in interacting proteins which are differentially expressed in human coronary artery endothelial cells (HCAEC) and human internal thoracic artery endothelial cells (HITAEC), according to the Gene Ontology (Cellular Component, Molecular Function, and Biological Process), UniProtKB Keywords, Reactome, and Kyoto Encyclopedia of Genes and Genomes (KEGG) databases (analysed by the Database for Annotation, Visualization and Integrated Discovery (DAVID)).

Bioinformatic term	Total proteins	Percent from differentially expressed proteins	Fold enrichment	FDR-corrected *p* value
Cell cycle, mitotic (Reactome)	27	10.1	2.20	3.30×10^{-3}
DNA replication (Reactome)	22	8.2	5.30	3.50×10^{-8}
Signaling by NOTCH (Reactome)	21	7.9	4.00	5.10×10^{-6}
Pre-NOTCH transcription and translation (Reactome)	17	6.4	8.30	6.60×10^{-9}
Pre-NOTCH expression and processing (Reactome)	17	6.4	7.00	5.70×10^{-8}
Cell junction (GO CC)	14	5.2	2.50	2.70×10^{-2}
Cell surface interactions at the vascular wall (Reactome)	13	4.9	4.20	7.10×10^{-4}
Angiogenesis (GO BP)	10	3.7	2.90	3.60×10^{-1}
Adherens junction (GO CC)	8	3.0	3.40	8.90×10^{-2}
Positive regulation of endothelial cell migration (GO BP)	7	2.6	7.60	3.80×10^{-2}
Basement membrane (GO CC)	7	2.6	5.70	2.30×10^{-2}
Cell-matrix adhesion (GO BP)	7	2.6	4.70	2.50×10^{-1}
Cell-cell junction (GO CC)	7	2.6	2.80	2.10×10^{-1}
Elastic fibre formation (Reactome)	6	2.2	6.10	2.40×10^{-2}
Positive regulation of endothelial cell proliferation (GO BP)	6	2.2	5.80	2.40×10^{-1}
Positive regulation of nitric-oxide synthase biosynthetic process (GO BP)	4	1.5	16.2	1.50×10^{-1}
Positive regulation of blood vessel endothelial cell migration (GO BP)	4	1.5	5.90	7.20×10^{-1}
Endothelial tube morphogenesis (GO BP)	3	1.1	24.2	3.30×10^{-1}
Establishment of endothelial barrier (GO BP)	3	1.1	10.40	7.60×10^{-1}
Basement membrane organization (GO BP)	3	1.1	9.90	7.90×10^{-1}

Supplementary Table S35. Molecular categories enriched in interacting proteins which are differentially expressed in human coronary artery endothelial cells (HCAEC) and human internal thoracic artery endothelial cells (HITAEC), according to the Gene Ontology (Cellular Component, Molecular Function, and Biological Process), UniProtKB Keywords, Reactome, and Kyoto Encyclopedia of Genes and Genomes (KEGG) databases (analysed by stringApp within the Cytoscape framework).

Bioinformatic term	Total proteins	Percent from differentially expressed proteins	Background proteins	FDR-corrected *p* value
Anchoring junction (GO CC)	44	17.32	1325	5.86×10^{-7}
Cell-substrate junction (GO CC)	30	11.81	426	$+2.20 \times 10^{-11}$
Focal adhesion (GO CC)	29	11.42	416	5.88×10^{-11}
Positive regulation of cell adhesion (GO BP)	21	8.27	485	4.80×10^{-4}
Cell-cell junction (GO CC)	17	6.69	499	6.70×10^{-3}
Blood vessel morphogenesis (GO BP)	16	6.30	419	1.82×10^{-2}
Angiogenesis (GO BP)	15	5.91	325	5.10×10^{-3}
Cell surface interactions at the vascular wall (Reactome)	13	5.12	139	8.03×10^{-6}
Positive regulation of endothelial cell migration (GO BP)	9	3.54	107	3.00×10^{-3}
Tight junction (KEGG)	9	3.54	157	3.20×10^{-3}
Cell-matrix adhesion (GO BP)	9	3.54	136	1.28×10^{-2}
Signaling by NOTCH (Reactome)	9	3.54	204	2.39×10^{-2}
Endothelium development (GO BP)	8	3.15	98	8.50×10^{-3}
Adherens junction (GO CC)	8	3.15	177	3.38×10^{-2}
Basement membrane (GO CC)	7	2.76	98	7.40×10^{-3}
Elastic fibre formation (Reactome)	6	2.36	44	1.20×10^{-3}
VEGFA-VEGFR2 pathway (Reactome)	6	2.36	96	2.70×10^{-2}
Pre-NOTCH transcription and translation (Reactome)	5	1.97	62	2.36×10^{-2}
Cell adhesion mediated by integrin (GO BP)	5	1.97	42	3.08×10^{-2}
Positive regulation of nitric-oxide synthase biosynthetic process (GO BP)	4	1.57	18	1.83×10^{-2}

Supplementary Table S36. Molecular categories enriched in interacting proteins which are differentially expressed in human coronary artery endothelial cells (HCAEC) and human internal thoracic artery endothelial cells (HITAEC) cultured under laminar flow, according to

the Gene Ontology (Cellular Component, Molecular Function, and Biological Process), UniProtKB Keywords, Reactome, and Kyoto Encyclopedia of Genes and Genomes (KEGG) databases (analysed by the Database for Annotation, Visualization and Integrated Discovery (DAVID)).

**Bioinformatic term**	**Total genes**	**Percent from differentially expressed genes**	**Fold enrichment**	**FDR-corrected *p* value**
Angiogenesis (GO BP)	33	6.4	4.9	2.0×10^{-10}
Cell junction (UniProtKB Keywords)	32	6.2	2.9	1.80×10^{-6}
Focal adhesion (KEGG)	32	6.2	3.5	9.10×10^{-8}
Cell-cell adhesion (GO BP)	22	4.2	4.3	8.20×10^{-6}
Cell-matrix adhesion (GO BP)	19	3.7	6.6	1.70×10^{-7}
Positive regulation of angiogenesis (GO BP)	19	3.7	4.4	4.30×10^{-5}
Cell surface interactions at the vascular wall (Reactome)	15	2.9	2.7	2.90×10^{-2}
Gap junction (KEGG)	13	2.5	3.2	3.40×10^{-3}
Cell-cell junction (GO CC)	13	2.5	2.7	2.20×10^{-2}
Elastic fibre formation (Reactome)	10	1.9	5.7	2.30×10^{-3}
Basement membrane (GO CC)	10	1.9	4.2	5.40×10^{-3}
Positive regulation of endothelial cell proliferation (GO BP)	10	1.9	5.1	5.50×10^{-3}
Positive regulation of cell adhesion (GO BP)	9	1.7	5.8	5.50×10^{-3}
Molecules associated with elastic fibres (Reactome)	9	1.7	6.1	3.50×10^{-3}
Bicellular tight junction (GO CC)	8	1.5	2.5	2.20×10^{-1}
Positive regulation of endothelial cell migration (GO BP)	8	1.5	4.5	3.70×10^{-2}
Notch signaling pathway (GO BP)	8	1.5	2.6	2.90×10^{-1}
Positive regulation of vascular endothelial growth factor production (GO BP)	7	1.4	8.9	4.60×10^{-3}
Sprouting angiogenesis (GO BP)	7	1.4	7.8	7.50×10^{-3}
Nitric oxide stimulates guanylate cyclase (Reactome)	6	1.2	6.8	3.30×10^{-2}
Positive regulation of nitric oxide biosynthetic process (GO BP)	6	1.2	5.0	9.80×10^{-2}
Anchoring junction (GO CC)	5	1.0	4.5	1.40×10^{-1}
Elastic fiber assembly (GO BP)	5	1.0	15.8	6.70×10^{-3}
Nitric-oxide synthase binding (GO MF)	4	0.8	9.9	1.10×10^{-1}

Nitric oxide mediated signal transduction (GO BP)	4	0.8	7.6	1.60×10^{-1}
Establishment of endothelial barrier (GO BP)	4	0.8	7.2	1.80×10^{-1}
Regulation of nitric-oxide synthase activity (GO BP)	3	0.6	12.7	2.10×10^{-1}

Supplementary Table S37. Molecular categories enriched in interacting proteins which are differentially expressed in human coronary artery endothelial cells (HCAEC) and human internal thoracic artery endothelial cells (HITAEC) cultured under laminar flow, according to the Gene Ontology (Cellular Component, Molecular Function, and Biological Process), UniProtKB Keywords, Reactome, and Kyoto Encyclopedia of Genes and Genomes (KEGG) databases (analysed by stringApp within the Cytoscape framework).

**Bioinformatic term**	**Total genes**	**Percent from differentially expressed genes**	**Background genes**	**FDR-corrected *p* value**
Cell junction (GO CC)	153	7,23%	2115	3.52×10^{-28}
Anchoring junction (GO CC)	97	7,32%	1325	4.75×10^{-17}
Cell adhesion (GO BP)	91	9,43%	965	1.35×10^{-22}
Vasculature development (GO BP)	61	11,57%	527	1.73×10^{-18}
Cell-cell adhesion (GO BP)	57	10,52%	542	1.47×10^{-15}
Positive regulation of cell adhesion (GO BP)	47	9,69%	485	1.33×10^{-11}
Angiogenesis (GO BP)	43	13,23%	325	1.15×10^{-14}
Cell-cell junction (GO CC)	34	6,81%	499	2.94×10^{-5}
Focal adhesion (KEGG)	32	16,41%	195	3.24×10^{-13}
Positive regulation of cell-cell adhesion (GO BP)	30	9,32%	322	4.95×10^{-7}
Cell-substrate adhesion (GO BP)	25	12,82%	195	3.21×10^{-08}
Cell junction assembly (GO BP)	24	8,79%	273	2.80×10^{-5}
Positive regulation of angiogenesis (GO BP)	21	12,65%	166	7.48×10^{-7}
Cell-matrix adhesion (GO BP)	20	14,71%	136	1.89×10^{-7}
Cell surface interactions at the vascular wall (Reactome)	14	10,07%	139	1.60×10^{-3}
Gap junction (KEGG)	13	14,94%	87	1.36×10^{-5}
Positive regulation of endothelial cell proliferation (GO BP)	13	13,54%	96	1.30×10^{-4}
Positive regulation of endothelial cell migration (GO BP)	13	12,15%	107	3.40×10^{-4}
Basement membrane (GO CC)	11	11,22%	98	1.90×10^{-3}

Positive regulation of cell junction assembly (GO BP)	11	10,58%	104	3.50×10^{-3}
Elastic fibre formation (Reactome)	10	22,73%	44	6.70×10^{-5}
Endothelium development (GO BP)	10	10,20%	98	7.70×10^{-3}
Tight junction (KEGG)	10	6,37%	157	2.77×10^{-2}
Molecules associated with elastic fibres (Reactome)	9	24,32%	37	1.30×10^{-4}
Sprouting angiogenesis (GO BP)	9	15,52%	58	1.20×10^{-3}
Endothelial cell differentiation (GO BP)	8	9,76%	82	2.71×10^{-2}
Positive regulation of vascular endothelial growth factor production (GO BP)	7	24,14%	29	7.40×10^{-4}
Regulation of nitric-oxide synthase activity (GO BP)	7	17,50%	40	3.50×10^{-3}
Nitric oxide stimulates guanylate cyclase (Reactome)	6	27,27%	22	2.30×10^{-3}
Positive regulation of nitric oxide biosynthetic process (GO BP)	6	13,95%	43	2.07×10^{-2}
Adherens junction (KEGG)	6	8,70%	69	3.03×10^{-2}
Elastic fiber assembly (GO BP)	5	41,67%	12	1.20×10^{-3}
Vascular endothelial growth factor signaling pathway (GO BP)	5	23,81%	21	7.80×10^{-3}
Establishment of endothelial barrier (GO BP)	5	15,15%	33	3.22×10^{-2}
Nitric oxide mediated signal transduction (GO BP)	4	19,05%	21	3.99×10^{-2}

Supplementary Table S38. Molecular categories enriched in interacting proteins which are differentially expressed in human coronary artery endothelial cells (HCAEC) and human internal thoracic artery endothelial cells (HITAEC) cultured under static conditions, according to the Gene Ontology (Cellular Component, Molecular Function, and Biological Process), UniProtKB Keywords, Reactome, and Kyoto Encyclopedia of Genes and Genomes (KEGG) databases (analysed by the Database for Annotation, Visualization and Integrated Discovery (DAVID)).

**Bioinformatic term**	**Total genes**	**Percent from differentially expressed genes**	**Fold enrichment**	**FDR-corrected *p* value**
Cell adhesion (GO BP)	42	9.0	3.1	3.20×10^{-7}
Focal adhesion (KEGG)	29	6.2	3.4	3.00×10^{-6}
Cell junction (GO CC)	18	3.8	1.8	1.10×10^{-1}

Signaling by NOTCH (Reactome)	17	3.6	2.0	1.60×10^{-1}
Angiogenesis (GO BP)	16	3.4	2.6	3.70×10^{-2}
Positive regulation of angiogenesis (GO BP)	14	3.0	3.6	9.10×10^{-3}
Cell-matrix adhesion (GO BP)	12	2.6	4.6	5.00×10^{-3}
Cell-cell adhesion (GO BP)	12	2.6	2.6	1.30×10^{-1}
Gap junction (KEGG)	11	2.3	3.0	3.80×10^{-2}
Cell-cell junction (GO CC)	11	2.3	2.6	8.60×10^{-2}
Pre-NOTCH expression and processing (Reactome)	9	1.9	2.3	3.00×10^{-1}
Signaling by NOTCH4 (Reactome)	8	1.7	2.7	2.50×10^{-1}
Pre-NOTCH transcription and translation (Reactome)	8	1.7	2.4	3.20×10^{-1}
Positive regulation of cell adhesion (GO BP)	8	1.7	5.7	2.00×10^{-2}
Nitric oxide stimulates guanylate cyclase (Reactome)	8	1.7	10.0	7.00×10^{-4}
Basement membrane (GO CC)	8	1.7	3.7	5.50×10^{-2}
Elastic fibre formation (Reactome)	7	1.5	4.4	1.00×10^{-1}
Positive regulation of endothelial cell proliferation (GO BP)	6	1.3	3.4	3.30×10^{-1}
Molecules associated with elastic fibres (Reactome)	6	1.3	4.5	1.60×10^{-1}
Nitric oxide mediated signal transduction (GO BP)	4	0.9	8.4	1.70×10^{-1}

Supplementary Table S39. Molecular categories enriched in interacting proteins which are differentially expressed in human coronary artery endothelial cells (HCAEC) and human internal thoracic artery endothelial cells (HITAEC) cultured under static conditions, according to the Gene Ontology (Cellular Component, Molecular Function, and Biological Process), UniProtKB Keywords, Reactome, and Kyoto Encyclopedia of Genes and Genomes (KEGG) databases (analysed by stringApp within the Cytoscape framework).

**Bioinformatic term**	**Total genes**	**Percent from differentially expressed genes**	**Background genes**	**FDR-corrected *p* value**
Cell junction (GO CC)	109	5,15%	2115	2.80×10^{-12}
Anchoring junction (GO CC)	63	4,75%	1325	1.72×10^{-5}
Cell adhesion (GO BP)	58	6,01%	965	2.39×10^{-8}
Positive regulation of cell adhesion (GO BP)	51	10,52%	485	2.18×10^{-15}
Positive regulation of cell-cell adhesion (GO BP)	36	11,18%	322	2.16×10^{-11}

Focal adhesion (KEGG)	29	14,87%	195	6.45×10^{-12}
Angiogenesis (GO BP)	24	7,38%	325	9.83×10^{-5}
Cell-substrate junction (GO CC)	24	5,63%	426	4.40×10^{-3}
Cell-cell junction (GO CC)	24	4,81%	499	2.59×10^{-2}
Cell junction assembly (GO BP)	20	7,33%	273	6.10×10^{-4}
Cell-substrate adhesion (GO BP)	17	8,72%	195	3.50×10^{-4}
Cell-matrix adhesion (GO BP)	14	10,29%	136	3.90×10^{-4}
Positive regulation of angiogenesis (GO BP)	14	8,43%	166	2.20×10^{-3}
Signaling by NOTCH (Reactome)	13	6,37%	204	2.70×10^{-2}
Gap junction (KEGG)	11	12,64%	87	1.80×10^{-4}
Basement membrane (GO CC)	11	11,22%	98	1.90×10^{-3}
Endothelium development (GO BP)	10	10,20%	98	4.90×10^{-3}
Nitric oxide stimulates guanylate cyclase (Reactome)	8	36,36%	22	2.42×10^{-5}
Signaling by NOTCH4 (Reactome)	8	9,88%	81	1.93×10^{-2}
Endothelial cell differentiation (GO BP)	8	9,76%	82	2.02×10^{-2}
Elastic fibre formation (Reactome)	7	15,91%	44	6.10×10^{-3}
Molecules associated with elastic fibres (Reactome)	6	16,22%	37	1.20×10^{-2}
Sprouting angiogenesis (GO BP)	6	10,34%	58	4.80×10^{-2}
Regulation of nitric oxide mediated signal transduction (GO BP)	4	40,00%	10	5.50×10^{-3}
Nitric oxide mediated signal transduction (GO BP)	4	19,05%	21	3.60×10^{-2}

Supplementary Table S40. Sequences of primers used for RT-qPCR.

Gene	Forward primer	Reverse primer
VCAM1	5′-CGTCTTGGTCAGCCCTTCCT-3′	5′-ACATTCATATACTCCCGCATCCTTC-3′
ICAM1	5′-TTGGGCATAGAGACCCCGTT-3′	5′-GCACATTGCTCAGTTCATACACC-3′
SELE	5′-ACCCTGGCTTCAGTGGACTC-3′	5′-TGCTTGGCAGGTAACCCCTAT-3′
SELP	5′-ATGGGTGGGAACCAAAAAGG-3′	5′-GGCTGACGGACTCTTGATGTAT-3′
IL6	5′-GGCACTGGCAGAAAACAACC-3′	5′-GCAAGTCTCCTCATTGAATCC-3′
CXCL8	5′-CAGAGACAGCAGAGCACAC-3′	5′-AGTTCTTTAGCACTCCTTGGC-3′
CCL2	5′-TTCTGTGCCCTGCTGCTCATAG-3′	5′-AGGTGACTGGGGCATTGATTG-3′
CXCL1	5′-GCTTGCCTCAATCCTGCATCC-3′	5′-ACAATCCAGGTGGCCTCTGC-3′

MIF	5′-GGTGTCCGAGAAGTCAGGCA-3′	5′-GGGGCACGTTGGTGTTTACG-3′
KLF2	5′-CAGCACTGGTCTGGTTGCTTG-3′	5′-ACCCACTGCACACGATGCTT-3′
KLF4	5′-GAAAAGGACCGCCACCCACA-3′	5′-AGCGGGCGAATTTCCATCCA-3′
NFE2L2	5′-GCACATCCAGTCAGAAACCAGT-3′	5′-ACTGAAACGTAGCCGAAGAAAC-3′
SNAI1	5′-CAGACCCACTCAGATGTCAAGAA-3′	5′-GGGCAGGTATGGAGAGGAAGA-3′
SNAI2	5′-ACTCCGAAGCCAAATGACAA-3′	5′-CTCTCTCTGTGGGTGTGTGT-3′
TWIST1	5′-GTCCGCAGTCTTACGAGGAG-3′	5′-GCTTGAGGGTCTGAATCTTGCT-3′
ZEB1	5′-GATGATGAATGCGAGTCAGATGC-3′	5′-ACAGCAGTGTCTTGTTGTTGT-3′
CDH5	5′-AAGCGTGAGTCGCAAGAATG-3′	5′-TCTCCAGGTTTTCGCCAGTG-3′
CDH2	5′-GCTTCTGGTGAAATCGCATTA-3′	5′-AGTCTCTCTTCTGCCTTTGTAG-3′
NOS3	5′-CCCGTTATCCCAGCTCCAAT-3′	5′-GTTGATGGCTTGACGTGCGTA-3′
GAPDH	5′-AGCCACATCGCTCAGACAC-3′	5′-GCCCAATACGACCAAATCC-3′
ACTB	5′-CATCGAGCACGGCATCGTCA-3′	5′-TAGCACAGCCTGGACAGCAAC-3′
B2M	5′-TCCATCCGACATTGAAGTTG-3′	5′-CGGCAGGCATACTCATCTT-3′
